# The impact of baseline health factors on second primary cancer risk after radiotherapy for prostate cancer

**DOI:** 10.2340/1651-226X.2024.24334

**Published:** 2024-06-30

**Authors:** Marie-Christina Jahreiß, Luca Incrocci, Katja K.H. Aben, Kim C. de Vries, Mischa Hoogeman, Maartje J. Hooning, Wilma D. Heemsbergen

**Affiliations:** a Department of Radiation Oncology, Erasmus MC Cancer Institute, Rotterdam, The Netherlands; b Department of Research, Netherlands Comprehensive Cancer Organization, Utrecht, The Netherlands; c Department for Health Evidence, Radboudumc, Nijmegen, The Netherlands; d Department of Medical Oncology, Erasmus MC Cancer Institute, Rotterdam, The Netherlands

**Keywords:** Comorbidities, smoking, second primary cancer, prostate cancer, radiotherapy

## Abstract

**Purpose:**

In evaluating second primary cancers (SPCs) following External Beam Radiotherapy (EBRT), the role of lifestyle factors is frequently not considered due to data limitations. We investigated the association between smoking, comorbidities, and SPC risks within EBRT-treated patients for localized prostate cancer (PCa).

**Patients & Methods:**

The study included 1,883 PCa survivors aged 50–79, treated between 2006 and 2013, with intensity-modulated radiotherapy (IMRT) or three-dimensional conformal radiotherapy (3D-CRT). Clinical data were combined with SPC and survival data from the Netherlands Cancer Registry with a 12-month latency period. Standardized Incidence Ratios (SIRs) were calculated comparing the EBRT cohort with the general Dutch population. To explore the effect of patient and treatment characteristics on SPCs we conducted a Cox regression analysis. Lastly, we estimated cumulative incidences of developing solid SPC, pelvis SPC, and non-pelvis SPC using a competing risk analysis.

**Results:**

Significantly increased SIRs were observed for all SPC (SIR = 1.21, 95% confidence interval [CI]: 1.08–1.34), pelvis SPC (SIR = 1.46, 95% CI: 1.18–1.78), and non-pelvis SPC (SIR = 1.18, 95% CI [1.04–1.34]). Smoking status was significantly associated with pelvic and non-pelvic SPCs. Charlson comorbidity index (CCI) ≥ 1 (Hazard Ratio [HR] = 1.45, 95% CI: 1.10–1.91), cardiovascular disease (HR = 1.41, 95% CI: 1.05–1.88), and chronic obstructive pulmonary disease (COPD) (HR = 1.91, 95% CI: 1.30–2.79) were significantly associated with non-pelvis SPC. The proportion of active smoking numbers in the cohort was similar to the general population.

**Interpretation:**

We conclude that the presence of comorbidities in the EBRT population might be a relevant factor in observed excess non-pelvis SPC risk, but not for excess pelvis SPC risk.

## Introduction

The development of a Second Primary Cancer (SPC) represents a multifaceted process influenced by a complex interplay of genetic, environmental, lifestyle, and treatment-related factors [1]. For prostate cancer (PCa) survivors specifically, significant advances in the detection and treatment have led to high overall survival rates [2]. Consequently, the consideration of adverse events such as the development of a radiation-induced SPC has become increasingly relevant.

One of the main treatment approaches for localized PCa patients is External Beam Radiotherapy (EBRT). EBRT particularly is the preferred choice of treatment for patients who may not be suitable candidates for radical prostatectomy due to physical fitness constraints [3, 4]. Furthermore, recent advances in the field of EBRT have contributed to its growing popularity, driven by the advantages it offers, such as reduced risk of urinary incontinence and diminished chances of erectile dysfunction when contrasted with radical prostatectomy [5].

Studies, however, have reported elevated risk for SPC development after EBRT as opposed to alternative treatments, such as radical prostatectomy [6–10]. This heightened risk can be attributed to the well-known fact that DNA damage in healthy tissue exposed to (very) low to intermediate radiation doses during EBRT may lead years later to the formation of cancerous cells [11]. Furthermore, hypotheses are made that this may also be attributed to the relatively less fit patient demographic eligible for EBRT [8, 12, 13]. It is thought that the excess SPC risk observed in patients receiving EBRT may not be solely attributed to radiation exposure, but could be influenced by unhealthy lifestyle choices, particularly, smoking status at time of treatment and the presence of comorbidities.

When exploring SPC risk after EBRT, large patient populations with sufficient follow-up are required. Cohort studies investigating the risk of SPC frequently rely on registry data, which typically lack comprehensive information on lifestyle-related variables. Hence, the primary objective of this study is to investigate the risk of SPCs following EBRT for localized PCa, considering lifestyle-related factors, particularly the smoking status at the time of treatment and the presence of comorbidities.

## Method

### Study design and participants

In this retrospective cohort investigation, a total of 1,883 survivors of localized PCa who had previously received EBRT at the Erasmus Medical Center in Rotterdam, The Netherlands, during the period spanning 2005–2013, were enrolled. The study cohort included individuals aged between ≥ 50 and < 80 years at the time of their treatment. EBRT was delivered using either three-dimensional radiotherapy (3D-CRT) or intensity-modulated radiotherapy (IMRT), with the transition to IMRT occurring gradually between 2007 and 2010. Exclusions from the study were made for individuals with metastatic disease at diagnosis, a history of prior pelvic EBRT, or those concurrently undergoing treatment for other malignancies. The study protocol received approval from the Medical Ethical Committee of the Erasmus Medical Center (EMC 1812730), and retrospective anonymized data collection was conducted in adherence to local and national regulations.

### Radiotherapy protocol

PCa treatment involved a prescribed dose of either 72 Gy or 78 Gy, delivered in daily 2-Gy fractions. From June 2010 onwards, the standard dose for intermediate to high-risk disease was 78 Gy. The dose to the seminal vesicles (SV) ranged from 0 to 78 Gy, based on SV involvement probability and evolving guidelines. 3D-CRT utilized a 3-field technique, while IMRT used a 7-field technique. 3D-CRT was delivered using either 18 megavoltage (MV) or 23 MV, whereas IMRT was delivered using either 10 MV or 18 MV. The planning target volume included the prostate (+/- SV) with a 10 mm or 5–7 mm margin, when offline or online setup verification and correction were used, respectively. Offline setup verification and correction relied on bony anatomy verified during the first three to four fractions and weekly afterwards, while online verification involved daily correction using implanted fiducial markers [14]. The setup verification employed planar MV imaging or a combination of planar MV and kilovoltage (kV) imaging for 10 MV IMRT. Larger pelvic imaging fields were occasionally used for offline MV imaging when the treatment fields lacked sufficient information. The recommended course of adjuvant hormonal therapy (ADT) typically spanned 36 months, although a small subset may have undergone a shorter ADT regimen (3 months). For this analysis, the brief ADT duration of 3 months was regarded as equivalent to no ADT.

### Data collection

Data were collected from electronic patient files, radiotherapy systems at Erasmus MC, and the Netherlands Cancer Registry (NCR). The collected information included patient and tumor characteristics, smoking status, comorbidity score (Charlson Comorbidity Index Items – CCI), adjuvant hormonal therapy (ADT) prescription, and details of the radiotherapy course. Details on SPC diagnosis, vital status, date of death, and emigration were obtained from the NCR using linkage based on the date of birth and postal code.

### Statistical analysis

Time at risk for developing a SPC was defined as >1 year after start of EBRT. The following cancer sites were evaluated: (a) all solid malignant cancers (C00-C80) (except for skin cancer, PCa, and mesothelioma), (b) solid pelvic SPC, (c) solid non-pelvic SPC, (d) solid cancers per anatomic region. We calculated Standardized Incidence Ratios (SIRs) to assess the risk of developing SPCs in the PCa survivors cohort compared to the Dutch general population. The SIR was determined by dividing the observed SPC cases in our study by the expected SPC cases in the Dutch general population. The expected cases in the Dutch general population were calculated by determining the expected number of first incident cancer. These analyses were based on age, gender, and calendar-specific cancer incidence rates obtained from the NCR. We considered all subsequent SPCs occurring after the PCa diagnosis up to December 31, 2021. SAS version 9.2 was used for these analyses. To explore the effect of patient-related and treatment-related characteristics on SPC development we conducted a Cox regression analysis, censoring for end of follow-up, death, or competing SPC endpoints, whichever came first. We considered the Cox regression model as a superior choice to evaluate risk factors in the presence of competing risks, as recommended by Alison [15]. Only the first SPC after PCa diagnosis was considered, and the main endpoints were: all solid SPC, all pelvis SPC, and all non-pelvis SPC. Baseline models were adjusted for age at radiotherapy and radiotherapy cohort, and statistical significance was set at *p* < 0.05. Additionally, we estimated cumulative incidences of developing all solid SPC, all pelvis SPC and non-pelvis SPC, using the Fine and Gray method [16]. Death and the development of a hematological SPC were considered as competing risks. Follow-up duration was defined from the start of radiotherapy until the date of SPC diagnosis, date of death, emigration, or end of follow-up or study (whichever occurred first). To account for differences in maximum follow-up between the two groups, all patients were censored after 14 years of follow-up, as IMRT was introduced later. Lastly, we explored the association between smoking status and the presence of comorbidities through a cross-tabulation analysis and assessed its statistical significance using a Chi-square test. The Cox regression analysis was carried out using SPSS software (Version 28, IBM Corporation, Armonk, NY, USA) and the competing risk analysis was conducted using Stata software (Version 14).

## Results

### General

The median follow-up period for the entire EBRT cohort (*N* = 1,883) was 8.9 years (Interquartile range (IQR): 5.9–11.6). The median age was 70 years (IQR: 65–74). Smoking status at time of treatment varied, with 30.3% categorized as never smokers, 23.8% as previous smokers, and 16.4% as active smokers, while 29.5% had unreported smoking status (Table 1). The CCI score ranged from 0 to 3+, with half of the population having a score of ≥1 (51%). Table 1 further includes information on the scored CCI items diabetes (present in 14%), cardiovascular disease (27%), previous cancer diagnosis (8%), and chronic obstructive pulmonary disease (COPD) (10%). Approximately 34% received a diagnosis of low to intermediate-grade PCa. 3D-CRT was administered to 45.3% of patients, while the rest received IMRT (54.7%).

**Table 1:** Baseline characteristics of the PCa patient cohort receiving EBRT.

Characteristics	EBRT Cohort (*N* = 1883)	
	N	%
**Patient characteristics**		
**Age at radiotherapy**		
50–69	860	45.7
70–79	1023	54.3
**Smoking status at time of EBRT**		
Never smoker	570	30.3
Previous smoker	449	23.8
Active smoker	308	16.4
Not reported	556	29.5
**Charlson comorbidity score**		
0	917	48.7
1	533	28.3
2	255	13.5
≥ 3	178	9.5
**Diabetes**		
No	1616	85.8
Yes	267	14.2
**(Previous) cardiovascular disease**		
No	1372	72.9
Yes	511	27.1
**Previous cancer diagnosis**		
No	1736	92.2
Yes	147	7.8
**COPD**		
No	1702	90.4
Yes	181	9.6
**Disease characteristics**		
**Risk group PCa**		
Low/intermediate	695	36.9
High	1188	63.1
**Treatment characteristics**		
**Technique**		
3D-CRT	853	45.3
IMRT	1031	54.7
**Dose prostate**		
72 Gy	400	21.3
78 Gy	1483	78.7
**Dose seminal vesicles**		
0 Gy	357	19.0
≥ 50 Gy	1526	81.0
**ADT 36 months**		
No	897	47.6
Yes	986	52.4
**EBRT cohort**		
2006–2010	955	50.7
2011–2015	928	49.3

### SPC risk in EBRT cohort versus general population

The estimated SIR for any SPC (excluding skin, mesothelioma, and prostate) was 1.21 (95% CI: 1.08–1.34) in the complete EBRT cohort. Increased SPC risks were observed for second solid cancers (SIR = 1.22, 95% CI: 1.11–1.39), second non-pelvis cancers (SIR = 1.18, 95% CI: 1.04–1.34), and second pelvis cancers (SIR = 1.46, 95% CI: 1.18–1.78) (Table 2). Patients aged 50–69 years had a higher risk of developing second non-pelvis cancers (SIR = 1.38, 95% CI: 1.14–1.66), while patients aged 70–79 years had an increased risk for second pelvis cancers (SIR = 1.49, 95% CI: 1.14–1.92).

**Table 2:** Standardized Incidence Ratios (SIRs), and Absolute Excess Risk (AER) for the complete EBRT cohort compared to the general Dutch male population, adjusted for age and calendar year.

Second tumor site	Complete EBRT Cohort (*N* = 1883)			
	Observed (n)	Expected (n)	SIR (95% CI)	AER
**All SPC**	355	294.5	**1.21 (1.08–1.34)**	42.74
**Solid**	328	263.7	**1.24 (1.11–1.39)**	45.06
**Haematological**	35	34.5	1.01 (0.71–1.41)	0.30
**Non-pelvis**	242	204.9	**1.18 (1.04–1.34)**	25.41
**Neck and up**	24	18.7	1.28 (0.82–1.91)	3.48
**Chest**	99	83.0	1.19 (0.97–1.45)	10.59
Lung & bronchus	69	65.1	1.06 (0.82–1.34)	2.57
Oesophagus	19	12.0	1.59 (0.95–2.47)	4.64
**Abdomen**	102	87.7	1.16 (0.95–1.41)	9.64
Stomach	4	6.6	0.61 (0.17–1.55)	-1.69
Colon	45	42.8	1.05 (0.77–1.41)	1.47
Pancreas	13	11.0	1.18 (0.63–2.02)	1.32
Kidney, renal pelvis & ureter	22	16.0	1.38 (0.86–2.08)	3.98
**Pelvis**	96	65.7	**1.46 (1.18-1.78)**	20.40
Bladder & urethra	68	45.1	**1.51 (1.17–1.91)**	15.34
Rectum & rectosigmoid	29	19.1	**1.52 (1.02–2.18)**	6.56
**Other**				
CNS	6	3.8	1.58 (0.58–3.44)	1.45
Unknown	8	6.7	1.20 (0.52–2.35)	0.87
**Age groups**				
**Patients aged 50–69 years**				
All SPC	155	115.0	**1.35 (1.14–1.58)**	57.21
Non-pelvis	112	81.2	**1.38 (1.14–1.66)**	42.82
Pelvis	35	24.8	1.41 (0.98–1.96)	13.84
**Patients aged 70–79 years**				
All SPC	200	179.5	1.11 (0.97–1.28)	28.63
Non-pelvis	130	123.7	1.05 (0.88–1.25)	8.53
Pelvis	61	40.9	**1.49 (1.14–1.92)**	26.84

*Significant SIRs are depicted in bold.

Observed and expected reflect number of observed and expected survivors experiencing the SPC event of interest. For SPC sub-sites, only the first experienced SPCs are taken into consideration. SIR = observed/expected. Evaluation period is diagnoses + 1 year up to end of exposure (end of observation period is 31-12-2021).

### Baseline health factors and SPC risk

**Figure 1:**
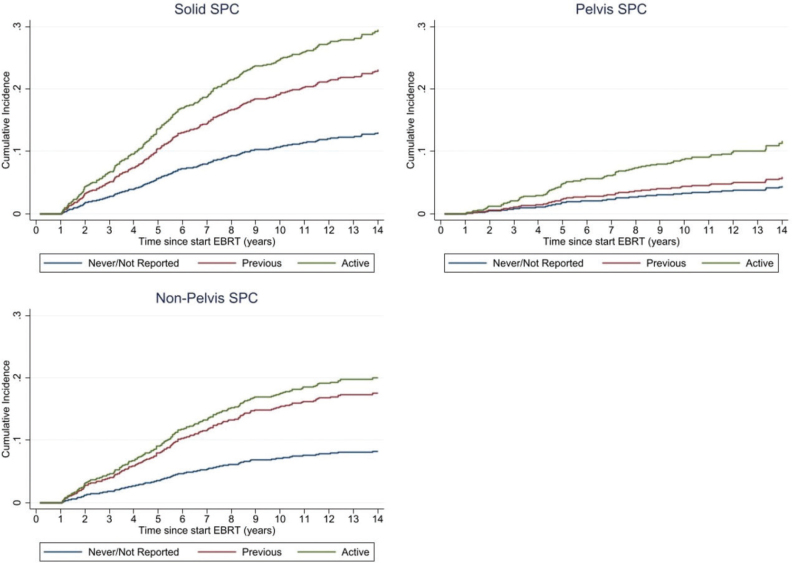
Cumulative incidence of solid SPC, pelvis SPC, and non-pelvis SPC stratified by smoking status at time of PCa treatment as estimated by the cumulative incidence function of the Fine and Gray model.

Table 3 presents the results of the Cox regression analysis for the development of solid SPC, pelvis SPC, and non-pelvis SPC in PCa patients treated with EBRT. Among patient-related factors, active smokers had a significantly increased risk for solid SPC (Hazard ratio (HR) = 2.86, 95% CI: 2.16–3.78, *p* < 0.001), pelvis SPC (HR = 3.15, 95% CI: 1.92–5.16, *p* < 0.001), and non-pelvis SPC (HR = 2.94, 95% CI: 2.08–4.17, *p* < 0.001) compared to never smokers or those with unreported smoking status (Table 3). Previous smokers had a significant increased risk for solid SPC (HR = 1.96, 95% CI: 1.51–2.57, *p* < 0.001), and non-pelvis SPC (HR = 2.36, 95% CI: 1.72–3.25, *p* < 0.001). Figure 1 depicts the cumulative incidences of SPC for the different SPC endpoints stratified by smoking status. CCI score of ≥1 was a significant predictor for second solid cancers (HR = 1.29, 95% CI: 1.03–1.62, *p* = 0.026), and second non-pelvis cancers (HR = 1.45, 95% CI: 1.10–1.91, *p* = 0.009). COPD and a history of cardiovascular disease were significant predictors for the development of second solid cancers overall, due to their impact on the development of second non-pelvis cancers. We furthermore found smoking status to be significantly associated with the presence of comorbidities (*p* < 0.001), such as COPD (*p* < 0.001), and a history of cardiovascular disease (*p* < 0.001). Other factors such as diabetes, previous cancer diagnosis, PCa risk group, EBRT technique, and ADT did not show statistically significant associations with SPC development (Table 3).

**Table 3:** Results of Cox regression analysis for solid SPC, pelvis SPC, and non-pelvis SPC development for PCa patients treated with EBRT: baseline model (adjusted for age and EBRT cohort).

Complete EBRT Cohort (*N* = 1883)							
Variable	Category	Endpoint: Solid SPC		Endpoint: Pelvis SPC		Endpoint: Non-Pelvis SPC	
		HR (95% CI)	*p*-value	HR (95% CI)	*p*-value	HR (95% CI)	*p*-value
**Patient-related**							
**Smoking at treatment**	Never smoker/Not reported (ref)	1	-	1	-	1	-
	Previous smoker	1.96 (1.51–2.57)	**< 0.001**	1.39 (0.82–2.38)	0.225	2.36 (1.72-3.25)	**< 0.001**
	Active smoker	2.86 (2.16–3.78)	**< 0.001**	3.15 (1.92–5.16)	**< 0.001**	2.94 (2.08-4.17)	**< 0.001**
**CCI Score**	0 (ref)	1	-	1	-	1	-
	≥1	1.29 (1.03–1.62)	**0.026**	0.97 (0.64–1.48)	0.886	1.45 (1.10-1.91)	**0.009**
	≥2	1.21 (0.93–1.57)	0.161	1.14 (0.69–1.88)	0.611	1.32 (0.97-1.81)	0.079
	≥3	1.59 (1.08–2.35)	**0.019**	1.03 (0.46–2.29)	0.946	1.98 (1.27-3.10)	**0.003**
**Diabetes**	No (ref)	1	-	1	-	1	-
	Yes	0.96 (0.69–1.34)	0.808	0.75 (0.38–1.50)	0.421	1.11 (0.75-1.64)	0.594
**Cardiovascular disease**	No (ref)	1	-	1	-	1	-
	Yes	1.28 (1.01–1.63)	**0.046**	0.93 (0.57–1.51)	0.771	1.41 (1.05-1.88)	**0.022**
**Previous cancer diagnosis**	No (ref)	1	-	1	-	1	-
	Yes	1.17 (0.76–1.79)	0.485	1.50 (0.72–3.13)	0.279	1.08 (0.64-1.84)	0.766
**COPD**	No (ref)	1	-	1	-	1	-
	Yes	1.72 (1.25–2.39)	**0.001**	1.24 (0.62–2.47)	0.541	1.91 (1.30-2.79)	**< 0.001**
**Disease-related**							
**Risk group PCa**	Low/intermediate risk (ref)	1	-	1	-	1	-
	High-risk	1.02 (0.81–1.29)	0.636	1.50 (0.95–2.36)	0.101	0.86 (0.65-1.14)	0.551
**Treatment-related**							
**EBRT technique**	3D-CRT (ref)	1	-	1	-	1	-
	IMRT	1.30 (0.93–1.82)	0.131	0.96 (0.50–1.84)	0.900	1.41 (0.93-2.13)	0.108
**ADT**	No (ref)	1	-	1	-	1	-
	Yes	0.98 (0.78–1.22)	0.826	1.42 (0.93–2.18)	0.107	0.78 (0.59-1.03)	0.081

*Significant *p*-values are depicted in bold.

## Discussion

In this study, we evaluated the risk of developing a SPC after EBRT for localized PCa, while taking into account lifestyle-related factors. The PCa EBRT population is partly an unhealthy selection of non-operable patients, and we hypothesized that smoking and comorbidity items might correlate with SPC patterns. The results of our study showed a significantly increased risk in the total cohort for SPC after EBRT compared to the Dutch general population in particular for pelvic SPC (SIR = 1.46), but also for non-pelvic SPC (SIR = 1.18). When we internally compared subgroups based on smoking and comorbidity profiles, we observed a substantial influence of smoking status at the time of treatment on SPC risk. We also found a significant association of the comorbidity COPD, and a history of cardiovascular disease on the risk of developing a non-pelvis SPC, but not for developing a pelvis SPC.

An important limitation of our study is the retrospective nature, which might have caused relevant underestimations of the presence of several evaluated factors. The proportion of active smokers within our study group (16%) aligns with the anticipated figures observed in the general population, as indicated by data from the Netherlands Expertise Centre for Tobacco Control [17]. Additionally, the overall study cohort did not exhibit an elevated risk of lung cancer when compared to the general population. This finding suggests that it is improbable that the count of active smokers is significantly underestimated. With respect to comorbidity, the observed numbers of COPD and diabetes cases are about the same as reported for the general population [18, 19], while we would expect higher numbers because of the unhealthy EBRT selection. Therefore, the retrospective nature of our study has likely caused underreporting. With respect to the CCI item ‘previous cancers’, the numbers are very accurate since we obtained them from the cancer registration. The number of patients with cardiovascular disease is probably fairly accurate since it is a factor that was often mentioned as a reason why a patient was non-operable.

The excess SPC risks we have identified in the entire EBRT cohort (compared to the general population) aligns with results reported in prior studies [6 – 8, 10, 12]. Consistent with previous research findings, we observed an increased probability of developing SPC in younger patients, except in cases of second cancers in the pelvis [6, 20, 21]. Those who were exposed to ionizing radiation at a younger age demonstrated greater vulnerability to its cancer-inducing effects in comparison to individuals exposed at later stages in life. The elevated risk of developing a second pelvis cancer in elderly patients is likely associated with an increased risk for second bladder cancer. Existing literature has established advanced age as a dominant risk factor for bladder cancer [22].

In a previous study, we reported that IMRT was associated with significantly increased risks of non-pelvis SPC (HR of 1.56, *p* = 0.034). In the current updated cohort this estimated effect was smaller and not significant anymore (HR of 1.41, *p* = 0.11) [20]. Our previous study results also suggested an interaction between technique and smoking status, with the highest non-pelvis SPC risk for the combination of active smoking and IMRT [20]. We have planned to study this phenomenon in more detail with additional dosimetry information once we have more follow-up available for the IMRT group, since it is known that radiation-induced SPCs risks are particularly increasing after 5–10 year. While it has long been established that smoking and radiation independently are risk factors for SPC development, the possible interplay between EBRT and smoking remains largely unexplored in existing literature. Boorjian reported from a large registry study in PCa patients that smoking alone has a relative risk of 2 for developing bladder cancer whereas for smoking plus EBRT this relative risk was doubled to about 4 [23]. In a recently conducted single-center study, we observed a significant increase in the risk of non-pelvis SPC among IMRT-treated patients versus 3D-CRT patients who smoked at the time of treatment [20], suggesting an interaction between active smoking and exposure to very low dose levels to larger body volumes during IMRT as opposed to 3DCRT. Smoking can sensitize tissues to the damaging effects of radiation, intensifying the cellular response to radiation-induced DNA damage [24].

Previous studies proposed that the increased risk of SPC observed in the group of patients who received EBRT might, to some extent, be associated with confounding variables, such as an unhealthy lifestyle [8, 12]. In the current study, we gathered comprehensive data regarding the presence of comorbidities among the study participants. The presence of comorbidities in a patient can provide indirect information about their lifestyle. Certain lifestyle choices like smoking, a sedentary lifestyle, poor dietary habits, obesity, and/or or excessive alcohol consumption can increase the risk of developing comorbidities such as cardiovascular disease, diabetes, or chronic respiratory conditions [25]. Our data analysis has verified an elevated risk of non-pelvis SPC in patients diagnosed with COPD and for those with a history of cardiovascular disease. The presence of comorbidities did not demonstrate a statistically significant association with an increased likelihood of SPC development in the pelvis. It is noteworthy to acknowledge that the majority of COPD cases, as well as cardiovascular cases, are connected to cigarette smoking or exposure to various forms of tobacco smoke [26, 27]. In our study, we also found a strong association between smoking and the presence of comorbidities, such as smoking and a history of cardiovascular disease.

The study’s strengths include its extensive sample size, prolonged follow-up duration, and comprehensive data on smoking status, comorbidities, and treatment specifics. Nevertheless, it is important to emphasize that these findings are derived from a single-center population, raising uncertainties about its generalizability. Hence, it is imperative to validate the results of this study by replicating them in an external study population for enhanced reliability and broader generalizability.

In conclusion, our study reaffirms an increased risk of SPCs in PCa patients undergoing EBRT and highlights the significant influence of smoking status on SPC risk. This underscores the importance of incorporating patients’ smoking habits into their overall risk assessment, particularly in the context of treatments like EBRT. By recognizing the impact of smoking, healthcare providers can customize patient care and actively support smoking cessation, ultimately improving the long-term health outcomes of cancer survivors. Future research should explore the potential interaction between smoking and EBRT in more detail. Moreover, our observed correlations with SPC endpoints suggest that comorbidities in the EBRT population are a dominant factor in the excess second cancers in the non-pelvis region, but not in the pelvis region.

## Data Availability

The raw data supporting the conclusions of this study will be made available by the authors, without undue reservation.
